# Place of death for people with HIV: a population-level comparison of eleven countries across three continents using death certificate data

**DOI:** 10.1186/s12879-018-2951-x

**Published:** 2018-01-25

**Authors:** Richard Harding, Stefano Marchetti, Bregje D. Onwuteaka-Philipsen, Donna M. Wilson, Miguel Ruiz-Ramos, Maria Cardenas-Turanzas, YongJoo Rhee, Lucas Morin, Katherine Hunt, Joan Teno, Cecilia Hakanson, Dirk Houttekier, Luc Deliens, Joachim Cohen

**Affiliations:** 1 King’s College London, Florence Nightingale Faculty of Nursing, Midwifery and Palliative Care, Cicely Saunders Institute, SE59PJ, London, UK; 2 Italian National Institute of Statistics, Rome, Italy; 3 Department of public and occupational health, VU University Medical Center, EMGO Institute for health and care research, Amsterdam, Netherlands; 4 University of Alberta, Edmonton, AB Canada; 5 Consejería de Igualdad, Salud y Políticas Sociales de Andalucía, Seville, Spain; 6 The University of Texas Health Science Center in Houston, Mac Govern Medical School, Houston, TX USA; 7 Dongduk Women’s University, Seoul, Korea; 8 Observatoire National de la Fin de Vie, Paris, France Ageing Research Center, Karolinska Institutet, Stockholm, Sweden; 9 Faculty of Health Sciences, University of Southampton, Southampton, UK; 10 Cambia Palliative Care Center of Excellence, University of Washington, Seattle, WA USA; 11 Department of Health Care Sciences, Palliative Research Centre, Ersta Sköndal University College, Stockholm, Sweden; 12 Department of Neurobiology, Care Science and Society, Karolinska Institutet, Stockholm, Sweden; 13 End-of-Life Care Research Group, Vrije Universiteit Brussel (VUB) & Ghent University, Brussels, Belgium; 14 Department of Medical Oncology, Ghent University Hospital, Ghent, Belgium

**Keywords:** HIV, Aids, End-of-life care, Place of death, Mortality

## Abstract

**Background:**

With over 1 million HIV-related deaths annually, quality end-of-life care remains a priority. Given strong public preference for home death, place of death is an important consideration for quality care. This 11 country study aimed to i) describe the number, proportion of all deaths, and demographics of HIV-related deaths; ii) identify place of death; iii) compare place of death to cancer patients iv), determine patient/health system factors associated with place of HIV-related death.

**Methods:**

In this retrospective analysis of death certification, data were extracted for the full population (ICD-10 codes B20-B24) for 1-year period: deceased’s demographic characteristics, place of death, healthcare supply.

**Results:**

i) 19,739 deaths were attributed to HIV. The highest proportion (per 1000 deaths) was for Mexico (9.8‰), and the lowest Sweden (0.2‰). The majority of deaths were among men (75%), and those aged <50 (69.1%). ii) Hospital was most common place of death in all countries: from 56.6% in the Netherlands to 90.9% in South Korea. The least common places were hospice facility (3.3%–5.7%), nursing home (0%–17.6%) and home (5.9%–26.3%).iii) Age-standardised relative risks found those with HIV less likely to die at home and more likely to die in hospital compared with cancer patients, and in most countries more likely to die in a nursing home. iv) Multivariate analysis found that men were more likely to die at home in UK, Canada, USA and Mexico; a greater number of hospital beds reduced the likelihood of dying at home in Italy and Mexico; a higher number of GPs was associated with home death in Italy and Mexico.

**Conclusions:**

With increasing comorbidity among people ageing with HIV, it is essential that end-of-life preferences are established and met. Differences in place of death according to country and diagnosis demonstrate the importance of ensuring a “good death” for people with HIV, alongside efforts to optimise treatment.

**Electronic supplementary material:**

The online version of this article (10.1186/s12879-018-2951-x) contains supplementary material, which is available to authorized users.

## Background

Despite the increasing availability of ART since 1996 [1] people still continue to die as a result of HIV infection [2]. This may be due to late presentation, limited access to treatment, suboptimal adherence, or HIV-related comorbidities and complications from long term infection and treatment, all of which require high quality HIV care through to the end of life [3]. Arguably, the advent of ART has led to a loss of focus on the end-of-life care needs of people living with HIV [4]. This gap is particularly concerning given clear evidence of continuing need for palliative and end-of-life care [5], and UNAIDS data estimating 1.2 million HIV-related deaths in 2014 [6].

The HIV epidemic is a challenge to health systems [7]. Even in high income countries with universal ART access, death rates among persons diagnosed with HIV infection remain high compared with the general population [8].

Place of death is a prime consideration for all people with serious illness. The clinical relevance lies in the potential for the patient to select place of death, thereby relieving concerns about the unknown process of dying, reduce anxieties about the future, enhance quality of life and the quality of death [9]. In European countries, the public express a strong preference to die at home [10], whereas evidence suggests that in low income countries the preference is for hospital death [11, 12]. This latter preference may reflect the variability of palliative and end-of-life care services to enable people to die at home [13], or reflect the lack of personal resources to deliver care in the home compared to hospital resources. It may also reflect the stigma associated with HIV [14], which may lead to social ostracisation and lack of support for home-based care.

Understanding where people die with HIV is important from a policy and funding perspective, as it indicates where resources and skills are needed to help patients and their families at the end of life. From a public health perspective, it is important to ensure well organized, high quality end-of-life care [15]. Place of death among people with HIV has not been studied since a single city Scottish study in 1995 [16]. Our cross-national study in 11 countries from three continents had the following objectives: i) to describe the number of HIV-related deaths, these as a proportion of all deaths, and demographics of the deceased; ii) identify place of death; iii) compare place of HIV-related death to those who died from cancer; iv), determine patient and health system factors associated with place of HIV-related death.

## Methods

### Study design

This study is part of the International Place of Death (iPoD) study, collecting death certificate data for the full population of deaths for a period of 1 year in 15 countries. Initial scoping of available data was conducted to ensure similarity of the variables. Data were obtained from 15 countries for the year 2008, except for the USA where 2007 was used as it was the most recent full available year, and partial data were obtained for Spain as Andalusia did not record place of death before 2010. All data were pooled into one common database for analysis.

Previous analyses from the iPoD study have reported place of death for cancer and non-cancer conditions [17–20], but have not reported HIV-related place of death. In addition to place of death data, we set out to obtain a number of clinical, sociodemographic and residential characteristics of the deceased, based on factors that have been identified as associated with the place of death [21].

### Participating countries

We conducted this analysis on countries with ART coverage of at least 80% for those eligible [22], and with known provision of palliative care according to the World Health Organisation’s global analysis [23]. The included countries were as follows: Canada, France, Italy, Belgium, UK, Sweden, USA (all categorized as having the highest palliative care provision ranking of “advanced integration into mainstream service provision”); Spain and the Netherlands (ranked by the WHO as having “preliminary integration”); and Mexico and South Korea (ranked as having “isolated provision”). Three countries were excluded in this analysis because of small numbers (i.e. <10 reported HIV-related deaths: Czech Republic, Hungary and New Zealand, see Table 1 below). A total of 11 countries (combining England and Wales) were included in the analyses.

**Table 1:** Deaths from HIV and healthcare supply density in 2008^a^ in 11 countries

Country	Abbreviations	Total number of deaths	Deaths from HIV	age				Urbanization
			N (proportion per 1000 deaths)	% male	% 0–49	% 50–69	% 70+	% living in core city / very strongly urbanized municipality^b^
France	FR	541,135	688 (1.3)	74.3	61.6	30.7	7.7	52.9
Italy	IT	578,192	1586 (2.7)	78.4	66.5	29.7	3.8	56.2
Spain (Andalusia)^a^	ES	57,380	188 (3.3)	84.6	76.1	21.8	2.1	68.6
Belgium	BE	102,924	50 (0.5)	70.0	64.0	24.0	12.0	80.0
The Netherlands	NL	135,136	53 (0.4)	73.6	56.6	37.7	5.7	81.1
Sweden	SE	91,471	18 (0.2)	72.2	22.2	77.8	0.0	93.3
England & Wales	UK	507,829	242 (0,5)	66.5	71.9	23.1	5.0	95.0
Canada	CA	182,134	319 (1.8)	77.1	65.2	30.7	4.1	88.7
United States^a^	US	2,428,342	11,332 (4.7)	71.7	63.0	34.6	2.4	/
Mexico	MX	528,093	5149 (9.8)	81.7	84.2	14.6	1.2	/
Korea	KR	247,757	99 (0.4)	89.9	60.6	31.3	8.1	28.3
Total		5,661,536	19,739 (3.5%)	75.0%	69.1%	28.5%	2.5%	**/**

Percentages may not add up to total due to rounding

^a^Reference year is 2007 in USA and 2010 in Spain;

^b^in Sweden and Canada, only urban and rural are distinguished

### Data sources

All included countries have similar death certification: an attending official certifies the sex of the deceased, diagnostic information and one or more primary and secondary causes of death, the day, and place of death. The respective authorities in each country check these certificates for inconsistencies and code the causes of death according to the WHO’s International Classification of Diseases 10th Revision (ICD-10) classification system of diseases [24]. All deaths with HIV/AIDS as an underlying cause of death (ICD-10 codes B20-B24) were selected.

Some death certificates contain additional socio-demographic information for the deceased, information often supplied by a civil servant of the civil registrar of the municipality of death; for other countries this information is not available through death certificates but unique linking with other databases (e.g. census data) allowed inclusion of a minimum number of socio-demographic variables. Additionally, available statistics about healthcare resources per capita were available and linked to the regions of residence in all countries. Details on variable data sources for each country are given in Table S1 (see Additional file 1), and data on UNAIDS prevalence estimates and ART coverage for advanced infection given in S2.

### Measures

The place of death was derived from the death certificate and included the following categories: “home”, “hospital”, “nursing home/care home” and “hospice facility” in all countries except for Mexico, where nursing home is not a recorded category as there are very few nursing home deaths, and hospice was only recorded in Netherlands, UK and USA.

As independent variables we included individual socio-demographic variables (age, sex), the clinical variable of cause of death (dying as a result of HIV/AIDS, rather than dying with HIV/AIDS) and residential characteristics and healthcare supply measures that have been identified as relevant in the literature on place of death of people with cancer [25]. Marital status was not used as a variable in this analysis due to the high prevalence of HIV among men who have sex with men and the rare legal provision for same sex marriage during the time period under analysis. The codes of the municipality/local authority of residence were matched with available data about urbanization level and healthcare supply. The healthcare supply data included the number of hospital beds/10,000 inhabitants, the number of GPs/10,000 inhabitants and the number of long-term care beds/1000 inhabitants aged 65 years or over, each per region of residence of the deceased. For reasons of data protection, in some countries the individual’s region of residence could not be obtained. For these countries, we used the nationally aggregated measure of healthcare supply.

### Statistical analysis

The analyses for objective i (i.e. to describe the number of HIV-related deaths and the demographics for these deaths, and these deaths as a proportion of total deaths), were conducted through descriptive analysis for each country separately.

For objective ii (i.e. to describe the place of death) and objective iii (i.e. to compare place of death to those who died from cancer), we conducted descriptive analysis by country and place of death. We then calculated the risk ratios (relative risks) by comparing the percentages for HIV with the age-standardized percentages for cancer, using direct standardization by applying the age distribution of the HIV deaths in each country to the cancer deaths. Age strata used for the standardization were as follows: 0–49, 50–59, 60–69, 70–79, 80–89, 90+. We selected cancer as the comparison group as it has been the diagnostic group that has the greatest coverage of palliative and end-of-life care. [26]

For objective iv (i.e. to determine whether there are any predictors of home death, independent variables of patient and health service characteristics) a parsimonious model was constructed. We selected the relevant variables for each country with stepwise backward variable selection into a logistic regression model. Only covariates with *p* < 0.05 were retained in the final model using the backward conditional procedure. We aimed for similar regression models across countries, in order to enhance comparability of the effects between countries. Statistical analyses were carried out using IBM SPSS version 21. Ethical approval was not required as we studied anonymised death certificate data.

## Results

Objective i) A total of 19,739 out of 5,661,536 deaths were directly attributed to HIV in the 11 included countries. These deaths accounted for 0.35% of all reported deaths (Table 1). The highest proportion (per 1000 deaths) of HIV-related deaths was in Mexico (9.8‰), and the lowest in Sweden (0.2‰). The majority of deaths were among men (75%), and those aged <50 (69.1%).

Objective ii) Hospital was the most common place of HIV-related death in all countries (ranging from 56.6% in the Netherlands to 90.9% in South Korea) (see Table 2). The least common places of HIV-related death were a hospice facility (range 3.3%–5.7%), nursing home (0%–17.6%) and home (range 5.9%–26.3%).

**Table 2:** The place of death of HIV vs cancer deaths in 14 countries during 2008

	Place of death	FR	IT	ES	BE	NL	SE	UK^a^	CA	US	MX	KR
	Number of deaths	688	1586	188	50	53	18	242	319	11,332	5149	99
		%	%	%	%	%	%	%	%	%	%	%
HIV	Home	15.8	9.2	9.6	18.0	15.1	5.9	12.0	12.9	14.9	26.3	9.1
	Hospital	79.9	84	85.1	76.0	56.6	76.5	78.1	73	62.8	71	90.9
	Nursing home	0.9	3.5	5.3	2.0	17.0	17.6	4.5	6.3	12.1	/^b^	0
	Hospice facility	/^b^	/^b^	/^b^	/^b^	7.5	/^b^	3.3	/^b^	5.7	/^b^	/^b^
	Others	3.3	3.4	0	4.0	3.8	0	2.1	7.8	4.6	2.8	0
Cancer (age-standardized)	Home	16.1	40.9	19.5	33.5	56.1	24.5	28.1	18.0	38.1	45.5	6.5
	Hospital	79.6	54.2	79.6	64.0	28.8	63.4	42.1	71.2	43.1	51.9	92.7
	Nursing home	1.3	1.6	1.0	1.8	7.6	10.8	2.2	5.9	7.5	/^b^	0.6
	Hospice facility	/^b^	/^b^	/^b^	/^b^	6.1	/^b^	25.3	/^b^	5.3	/^b^	/^b^
	Others	3.0	3.3	0.1	0.8	1.5	1.4	1.8	4.8	5.7	2.6	0.2
Relative Risks: (HIV vs Age-standardized percentages for cancer)	Home	0.98	0.22	0.49	0.54	0.27	0.24	0.43	0.72	0.39	0.58	1.39
	Hospital	1.00	1.55	1.07	1.19	1.97	1.21	1.85	1.03	1.45	1.37	0.98
	Nursing home	0.71	2.22	5.49	1.13	2.23	1.63	2.03	1.06	1.61	/^b^	/^b^
	Hospice facility	/^b^	/^b^	/^b^	/^b^	1.23	/^b^	0.13		1.08	/^b^	/^b^
	Others	1.09	1.03	0.00	5.18	0.50	0.00	1.00	1.63	0.81	1.06	0.00

^a^England and Wales

^b^Category not available on the death certificate data

Objective iii) After age-standardization, the relative risks indicate that people with HIV were less likely to die at home and more likely to die in hospitals compared with cancer patients (except in Korea); and in most countries (when reported) were also more likely to die in a nursing home. After age-standardization, people with HIV were considerably less likely than cancer patients to die in a hospice facility in UK, but slightly more likely in the USA.

Objective iv) The multivariable regression analyses revealed that for HIV-related deaths, males were more likely to die at home in UK, USA, Canada and Mexico (Table 3). Controlling for other confounders, we found no significant association with age. Educational attainment was associated with home death in Mexico and Spain; where those with higher education more likely to die in hospital. Persons with HIV living in more rural areas were less likely to die at home in Italy, but more likely in Spain compared to those living in urban areas. Living in a region with a higher number of hospital beds reduced the chances of dying at home in Italy and Mexico, and living in an area with a higher number of general practitioners increased the chances of dying at home in the UK and Mexico (but reduced them in the USA).

**Table 3:** Multivariable logistic regression models per country of factors associated with home death vs other (odds ratios and 95% Cis)

	FR	IT	ES	BE	NL	SE	UK	CA	US	MX	KR
Sex											
male (vs female)	ns	ns	ns	ns	ns	ns	3.59 (1.19–10.78)	3.10 (1.07–.01)	1.18 (1.05–.33)	1.23 (1.04–.46)	ns
Age											
0–49	ns	ns	ns	ns	ns	ns	ns	ns	ns	ns	ns
50–59	ns	ns	ns	ns	ns	ns	ns	ns	ns	ns	ns
60 and older	ns	ns	ns	ns	ns	ns	ns	ns	ns	ns	ns
Educational attainment											
primary or less	ns	ns	Ref cat	ns	NA	ns	NA	NA	ns	Ref cat	ns
lower secondary	ns	ns	0.31 (0.08–1.23)	ns	NA	ns	NA	NA	ns	0.59 (0.45–0.77)	NA
higher secondary	ns	ns	0.24 (0.05–1.05)	ns	NA	ns	NA	NA	ns	0.61 (0.51–0.72)	ns
higher	ns	ns	/	ns	NA	ns	NA	NA	ns	0.45 (0.37–0.53)	ns
Urbanization											
strong	ns	Ref cat	Ref cat	ns	ns	ns	ns	ns	NA	NA	ns
average	ns	0.31 (0.13–0.73)	1.22 (0.35–4.24)	ns	ns	ns	ns	ns	NA	NA	ns
rural	ns	0.57 (0.23–1.42)	21.09 (1.55–286.90)	ns	ns	ns	ns	ns	NA	NA	ns
LTC beds per 1000 65plus	ns	ns	ns	ns	ns	ns	ns	ns	0.99 (0.99–0.999)	ns	NA
Hospital beds per 10.000	1.03 (1.01–1.06)	0.88 (0.80–0.96)	ns	ns	ns	ns	ns	ns	ns	0.70 (0.64–0.75)	NA
GPs per 10.000	ns	ns	ns	ns	ns	ns	4.25 (1.29–13.98)	ns	0.93 (0.91–0.95)	1.20 (1.16–1.25)	NA

*Abbreviations*: *Ns* not significant in the logistic regression model and therefore omitted in the final model, *NA* not available, *Ref cat* reference category, *NA* not available in database (i.e. there are no long term care beds in Mexico)

There is no separate category lower secondary education for educational attainment in Korea. No regional data on hospital beds, LTC beds and GPs were available for Korea

## Discussion

This study found that the majority of people who died from HIV-related causes died in hospital (56.6–90.9%). After age standardisation, this rate is higher than for people who died from conditions indicative of palliative care need (i.e. cancer, renal failure, heart failure, liver failure, COPD, Alzheimer’s, Parkinson’s, Motor Neurone Disease, Huntingdon’s; 25–85%).

The higher proportion of hospital deaths for HIV-related causes may be due to the disproportionate number of men who have sex with men affected by HIV, who report poor communication by clinicians at end of life and disenfranchisement of their significant others [27, 28]. It is also possible that the trajectory to death is less predictable for people with HIV, which may trigger hospital admissions for potentially life-extending interventions compared to cancer patients [29]. It may also be partly explained by the admission and initiation of ART in hospital for people with late presentation of HIV, attempting new lines of therapy when a treatment regimen fails, or the complexity of prognostication for those who face clinical uncertainty. Also, as persons dying from HIV tend to be younger than those dying of other conditions (i.e. dementia [30], Parkinsons [31] Cancer [17] and other terminal conditions) there may be reluctance on the part of clinicians to discuss the end of life and options for place of death. However, we observed that even after age standardization, people dying of cancer in all countries except South Korea were more likely to die at home than those dying of HIV. That people dying of cancer are more able to die at home suggests they are potentially more able to receive formal and informal care in the home, which together enable home death [25]. This reflects the origins and persisting cancer focus of palliative care. We also note that people dying with HIV in the UK did not die in hospice facilities as often as those in the USA. HIV-related deaths in the UK were considerably less likely than cancer deaths to occur in a hospice facility. However, HIV and cancer deaths in the USA were more or less equally likely to occur in a hospice facility. This observation may reflect a specifically UK historical context, as the UK developed HIV hospice care outside of existing hospices [32], but HIV-specific hospices have now closed, leaving little experience of HIV care among mainstream hospice providers.

The use of death certificate data has the major advantage of providing population-level data that are not subject to potential bias inherent to sampling and that are cross-nationally comparable, as the procedure for registering deaths is similar across countries. Limitations of the present data are related to the type of information collected in the death certificate as it is not possible to ascertain preference for place of death, compare preferences among countries, or to infer how much time is spent in different care settings prior to death. The validity of the underlying cause of death as recorded on the death certificate has been contested [33], although the use of aggregated cause of death categories has most likely mitigated the risk of misclassification. However, HIV is likely underreported as the underlying cause of death [34]. We also acknowledge that the data source only allow us to identify people dying from, rather than with, HIV infection. Information on household characteristics and social support may have been a useful variable in our analysis. The variable that measured number of GPs/10,000 inhabitants is a useful measure of primary care coverage, but GP involvement in HIV care may vary between countries. For example, in the USA it is uncommon for HIV to be managed in primary care settings.

Although studies have demonstrated a preference among dying adults to die at home, the available data on these preferences has not been specific to preferences among people living with, or dying from, HIV. Evidence is not available on preference for place of death among people living with HIV, how this varies between countries, or how much time is spent at home prior to death. These should be priority areas of investigation.

It is important that policy and service configuration enable high quality end-of-life care in contexts of both clinical uncertainty and unavoidable death. Policy, funding, education and training are essential Governmental responsibilities in light of the World Health Assembly’s resolution on palliative care [35]. While we should not presume that those who die in hospital do not receive palliative care, evidence found that in France less than half of hospitalized patients in need of palliative care at the end of life actually received palliative care during their admission [36]. The barriers to palliative care for people with HIV have been well described, and include specific disease-related, clinician-related and service-related factors [37].

## Conclusions

People dying from HIV were, in all studied countries, most likely to die in hospital as compared to people dying of cancer. This indicates a particular challenge to provide high quality end-of-life care for those facing HIV-related death. Not enough is known about what is done clinically in hospitals to achieve a “good death” for people with HIV. Arguably, this lack of evidence on the quality of death also exists for deaths in nursing homes, hospices, or other places, notably the home. Except in Mexico, we found that less than a fifth of persons with HIV died at home. Local policy is likely to be highly influential, for example in Korea there are legal difficulties in providing medical services outside of hospital settings. This variability is also reflected in state and national laws on advance care plans, living wills and other relevant mechanisms. There is within-country variability (e.g. by State in the USA) and also between-country (for example in Germany home palliative care is a legal right; in the UK individuals have a legal right to appoint a proxy or make an advance directive) Further research is needed to determine end-of-life care preferences regarding care setting among people with HIV, on place of care in the last months of life, and the reasons why they do not spend their final hours or days at home. The availability of death registries in low income countries is also a future priority, so that those countries with poorest ART coverage can also study place of death. With increasing comorbidity among people ageing with HIV, it is essential that end-of-life care preferences are established, and that plans are made and carried out for those who prefer to die at home or in a hospice, or a hospital. In this study we have identified a high proportion of hospital deaths, and inequity in place of death. Policy makers must ensure that initiatives to achieve home death do not discriminate between conditions. Clinicians must ensure that while they encourage patients to “hope for the best”, they must also enable them to “plan for the worst”, and ensure that home death can be achieved irrespective of diagnosis. This new knowledge highlights the importance of ensuring a “good death” for people with HIV, alongside efforts to optimise treatment.

## Additional file

Additional file 1: Table S1. Supplementary Material for Place of death for people with HIV: a population-level comparison of eleven countries across three continents using death certificate data. About different data sources and the levels of linkage of data. **Table S2.** Descriptive data on HIV prevalence and ART coverage for each country, drawn from UNAIDS Global Report 2008. Description of HIV prevalence and ART coverage for each participating country. (DOCX 28 kb)

## Abbreviations

AIDS: Acquired Immune Deficiency Syndrome
ART: Antiretroviral Therapy
GP: General Practitioner
HIV: Human Immune Deficiency Virus
ICD: 10th revision of the International Statistical Classification of Diseases and Related Health Problems
UANIDS: Joint United Nations Programme on HIV/AIDS
WHO: World Health Organisation
