# Supplementary material for: Place of death for people with HIV: a population-level comparison of eleven countries across three continents using death certificate data
Source: BMC Infect Dis. 2018 Jan 25;18:55. doi: 10.1186/s12879-018-2951-x (PMC5785855; doi:10.1186/s12879-018-2951-x)
Supplement: Supplementary file 1 — Supplementary Material for Place of death for people with HIV: a population-level comparison of eleven countries across three continents using death certificate data. About different data sources and the levels of linkage of data. Table S2. Descriptive data on HIV prevalence and ART coverage for each country, drawn from UNAIDS Global Report 2008. Description of HIV prevalence and ART coverage for each participating country. (DOCX 28 kb) [file 12879_2018_2951_MOESM1_ESM.docx]

Supplementary Material for:

**Place of death for people with HIV: a population-level comparison of eleven countries across three continents using death certificate data**

R Harding, S Marchetti, B D Onwuteaka-philipsen, D M Wilson, M Ruiz-Ramos, M Cardenas-Turanzas,Y Rhee, L Morin, K Hunt, J Teno, C Hakanson, D Houttekier, L Deliens, J Cohen.

**Methods**

*About different data sources and the levels of linkage of data*

Information about the source of the healthcare resource statistics, the degree of urbanization and details of the level of linkage are provided in Table S1.

Table S1: Sources of the death certificate data, the data on healthcare supply, the ecological levels of the healthcare supply data linked to the individual death certificates, degree of urbanisation and population size per health region

|  | **Belgium** | **Canada** | **Czech Republic** | **England** | **France** |
| --- | --- | --- | --- | --- | --- |
| **Death certificate data** | Flemish Agency for Care and Health, Brussels Health and Social Observatory, French Community of Belgium, data for 2008 | Statistics Canada, data for 2008. Quebec death certificate data (managed by the Quebec province) are not integrated with the death certificate data of the rest of the country (managed by Statistics Canada). | Institute of Health Information and Statistics of the Czech Republic (UZIS), data for 2008 | Office for National Statistics (ONS), data for 2008 | Inserm-CépiDc (Centre d'épidémiologie sur les causes médicales de décès, Institut national de la santé et de la recherche médicale), data for 2008 |
| **Hospital beds** |  |  |  |  |  |
| source of data | Federal Public Service Health, Food Chain Safety And Environment, data for 2008 | Statistics Canada, data for 2008 | Institute of Health Information and Statistics of the Czech Republic (UZIS), data for 2008 | Department of Health (form KH03), data for 2008 | Ecosanté (institut de recherche et documentation en economie de la santé ), data for 2008 |
| area level of linkage | For Flanders and Brussels: health care region, large city level (14 regions); for Wallonia: province (5 regions, no health care regions exist in Wallonia) | Province (12 regions, excluding Quebec province) | Country-level | Strategic Health Authority (10 regions) | Administrative region (22 regions) |
| **LTC beds** |  |  |  |  |  |
| source of data | National institute for health and disability insurance (INAMI-RIZIV), data for 2008 | Statistics Canada, data for 2008 | Institute of Health Information and Statistics of the Czech Republic (UZIS), data for 2008 | Care Standards Act data (care homes active as of 31st March 2008) / CQC database) | Ecosanté (Institut de recherche et documentation en economie de la santé ) |
| area level of linkage | For Flanders and Brussels: Health Care Region, small city level (60 regions); for Wallonia: borough (arrondissement) (20 regions, no health care regions exist in Wallonia) | Province (12 regions, excluding Quebec province) | Country-level | Strategic health Authority (10 regions) | Administrative region (22 regions) |

|  | **Belgium** | **Canada** | **Czech Republic** | **England** | **France** |
| --- | --- | --- | --- | --- | --- |
| **General practitioners** |  |  |  |  |  |
| source of data | National institute for health and disability insurance (INAMI-RIZIV), data for 2008 | Statistics Canada, data for 2008 | Institute of Health Information and Statistics of the Czech Republic (UZIS), data for 2008 | Department of Health, data for 2008 | Direction de la recherche, des études, de l'évaluation et des statistiques (DREES) & Ministère des affaires sociales, de la santé et des droits des femmes (ADELI database), data for 2008 |
| area level of linkage | For Flanders and Brussels: Health Care Region, small city level (60 regions); for Wallonia: borough (arrondissement) (20 regions, no health care regions exist in Wallonia) | Province (12 regions, excluding Quebec province) | Country-level | Strategic health Authority (10 regions) | Administrative region (22 regions) |
| **Population size per health region** | Statistics Belgium, data for 2008 | Statistics Canada, data for 2008 | Czech Statistical Office, data for 2008 | Office for National Statistics (mid-2008 population estimates) | National Institute of Statistics and Economic Studies (INSEE), data for 2008 |
| **Degree of urbanisation** | Statistics Belgium (calculated at the municipality level) | Statistics Canada | n/a | n/a | National Institute of Statistics and Economic Studies (INSEE) |

|  | **Hungary** | **Italy** | **South Korea** | **Mexico** | **New Zealand** | |
| --- | --- | --- | --- | --- | --- | --- |
| **Death certificate data** | Central Statistical Office Hungary, data for 2008 | Italian National Institute of Statistics (Unit for Cause of Death Statistics), data for 2008 | Statistics Korea, data for 2008 | Secretaria de Salud, Sistema Nacional de Informacion en Salud (SINAIS)*, data for 2008 | New Zealand Ministry of Health, data for 2008 | |
| **Hospital beds** |  |  |  |  |  | |
| source of data | OECD, data for 2008 | Ministry of Health (Italian Health Care data), data for 2009 | OECD, data for 2008 | Secretaria de Salud, Sistema Nacional de Informacion en Salud (SINAIS)*, data for 2008 | New Zealand Ministry of Health, data for 2008 | |
| area level of linkage | Country-level | Macroregions (5 regions) | Country-level | Municipality (2456 municipalities) | District Health Board region (21 regions) | |
| **LTC beds** |  |  |  |  |  | |
| source of data | Hungarian Central Statistical Office, data for 2008 | Ministry of Health (Italian Health Care data), data for 2009 | OECD, data for 2008 | n/a | New Zealand Ministry of Health, data for 2008 | |
| area level of linkage | Country-level | Macroregions (5 regions) | Country-level |  | District Health Board region (21 regions) | |
| **GPs** |  |  |  |  |  | |
| source of data | Hungarian Central Statistical Office, data for 2008 | Ministry of Health (Italian Health Care data), data for 2009 | OECD, data for 2008 | Secretaria de Salud, Sistema Nacional de Informacion en Salud (SINAIS), data for 2008 (physicians of all specialties, including GPs) | New Zealand Ministry of Health, data for 2008 | |
| area level of linkage | Country-level | Macroregions (5 regions) | Country-level | Municipality (2456 municipalities) | District Health Board region (21 regions) | |
| **Population size per health region** | Hungarian Central Statistical Office, data for 2008 | National Institute of Statistics (Istat) | OECD, data for 2008 | Instituto Nacional de Estadistica Geografia  (INEGI) | New Zealand Ministry of Health, data for 2008 | |
| **Degree of urbanisation** | / | National Institute of Statistics (Istat) | Statistics Korea | n/a | n/a | |
|  |  |  |  | |  |  |

|  | **Spain (Andalusia)** | **The Netherlands** | **United States** | **Wales** |
| --- | --- | --- | --- | --- |
| **Death certificate data** | Instituto de Estadística y Cartografía de Andalucía, data for 2010. Place of death data were not available on death certificates for the whole of Spain at the time of data collection. | Netherlands: Statistics Netherlands (CBS), data for 2008 | Center for Disease Control and Prevention (CDC) based on Data Use Agreement, data for 2007 | Office for National Statistics (ONS), data for 2008 |
| **Hospital beds** |  |  |  |  |
| source of data | Andalusian Health Service (Servicio Andaluz de Salud), data for 2010 | Netherlands institute for health services research, data for 2008 | Statehealthfacts.org, data for 2007 | Statistics Wales, data for 2008 |
| area level of linkage | provinces (8 regions) | provinces (12 regions) | 50 states + District of Columbia | Primary Care Organizations (7 regions) |
| **LTC beds** |  |  |  |  |
| source of data | Andalusian Health Service (Servicio Andaluz de Salud), data for 2010 | Netherlands institute for health services research, data for 2008 | Statehealthfacts.org, data for 2007 | Care Standards Act data (care homes active as at 31st March 2008) / CQC database |
| area level of linkage | health districts (33 regions) | provinces (12 regions) | 50 states + District of Columbia | Country-level (no regional data available) |
| **GPs** |  |  |  |  |
| source of data | Andalusian Health Service (Servicio Andaluz de Salud), data for 2010 | Netherlands institute for health services research, data for 2008 | Statehealthfacts.org, data for 2007 (primary care physicians) | Statistics Wales, data for 2008 |
| area level of linkage | health districts (33 regions) | provinces (12 regions) | 50 states + District of Columbia | Primary Care Organizations (7 regions) |
| **Population size per health region** | Instituto Nacional de Estadisticas (Spanish Statistical Office), data for 2010 | Netherlands: Statistics Netherlands (CBS), data for 2008 | United States Census Bureau, data for 2007 | Statistics Wales, data for 2008 |
| **Degree of urbanisation** | Instituto Nacional de Estadisticas (Spanish Statistical Office) | Statistics Netherlands (CBS) | n/a | n/a |

Table S2 Descriptive data on HIV prevalence and ART coverage for each country, drawn from UNAIDS Global Report 2008 [1]

| **Data source** | **Death certificate** | **UNAIDS report 2008** | |
| --- | --- | --- | --- |
|  | **N deaths**  **(% per 1000 deaths)** | **Estimated number of adults & children living with HV** | **% with advanced infection on ART** |
| **Country** |  |  |  |
| France | 688 (1.3) | 140,000 | 64% |
| Italy | 1586 (2.7) | 150,000 | - |
| Spain (Andalusia)* | 188 (3.3) | 140,000 | - |
| Belgium | 50 (0.5) | 15,000 | 67% |
| The Netherlands | 53 (0.4) | 18,000 | - |
| Sweden | 18 (0.2) | 6,200 | 74% |
| England & Wales** | 242 (0,5) | 77,000 | >95% |
| Canada | 319 (1.8) | 73,000 | - |
| United States | 11332 (4.7) | 1,200,000 | - |
| Mexico | 5149 (9.8) | 200,000 | 54% |
| Korea | 99 (0.4) | 13,000 | - |

*UNAIDS data for whole of Spain

** UNAIDS data for United Kingdom

Reference

1. UNAIDS.: **Report on the Global HIV/AIDS Epidemic 2008**.
